# Impact of protein corona on magnetic particle spectroscopy-based bioassays

**DOI:** 10.1039/d5na01037c

**Published:** 2026-01-22

**Authors:** Gabrielle Moss, Christian Knopke, Solomon G. Diamond

**Affiliations:** a Thayer School of Engineering, Dartmouth College Hanover NH 03755 USA gabrielle.r.moss.th@dartmouth.edu; b Lodestone Biomedical LLC Lebanon NH 03766 USA

## Abstract

Magnetic nanoparticles (MNPs) can be functionalized with targeting ligands to give them an affinity for a biomolecule of interest. Functionalized MNPs (fMNPs) can aggregate in the presence of a multivalent target, causing a change in their magnetization. The 3rd harmonic phase of the fMNP magnetization signal can be proportional to the multivalent target concentration. When fMNPs are suspended in protein rich media (like biological fluids), off-target proteins tend to non-specifically adsorb to them, potentially masking their targeting ligands, and leading to a change in fMNP target affinity. The layer of adsorbed off-target proteins is commonly referred to as a protein corona. We used a model system consisting of biotinylated MNPs that target streptavidin to study the impact of protein corona formation on fMNP-based biosensing. Interestingly, the resolution of our biotinylated MNP-based aggregation assay changed from 64.43 nM streptavidin in the absence of off-target serum proteins, to 3.22 nM streptavidin in the presence of off-target serum proteins. Therefore, biotinylated MNP streptavidin sensitivity increased in the presence of off-target serum proteins. We attribute the increase in biotinylated MNP streptavidin sensitivity to competition between streptavidin and off-target serum proteins with a low biotin binding affinity. In contrast, competition between streptavidin and an off-target protein with a high biotin binding affinity decreased biotinylated MNP streptavidin sensitivity. Our results can be leveraged to inform the design optimization of an *in vivo* fMNP-based biosensor. Additionally, our results can also be leveraged to design an *in vitro* fMNP-based biosensor with a diluent off-target protein concentration and binding affinity optimal for target quantification in a tailored range.

## Abstract 2

We prepared a homogenous assay consisting of biotinylated magnetic nanoparticles that target streptavidin. Our assay's measurable concentration range shifted to lower streptavidin concentrations in the presence of low affinity off-target protiens.

## Graphical abstract

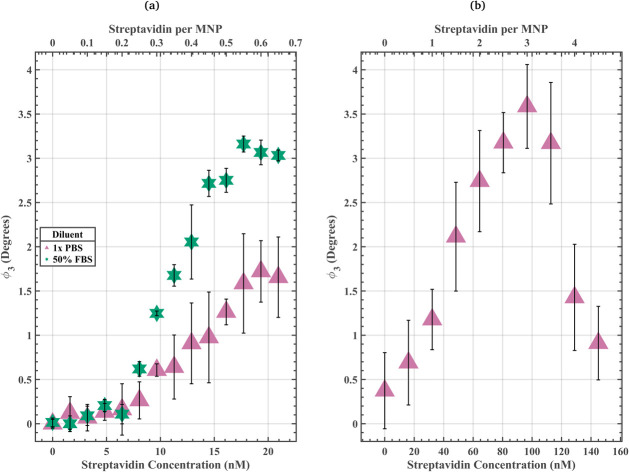

## 1 Introduction

Magnetic nanoparticle (MNP)-based aggregation assays have been engineered to sense clinically relevant biomolecules including H1N1 virus,^1^ thrombin,^2^ staphylococal toxins,^3^ and SARS-CoV2 spike and nucleocapsid proteins^4^*in vitro*. Wherein functionalized MNP (fMNP)-target binding is transduced *via* magnetic particle spectroscopy (MPS) and can be modeled *via* Brownian and Néel relaxation.^5,6^ It is desirable for an aggregation assay to be wash-free for more rapid results with simpler protocols. Wherein minimal sample manipulation is required prior to target biomolecule quantification. Sample manipulation prior to target quantification can rid the sample of off-target proteins that may interfere with the assay's detection element(s), leading to an inaccurate result. For fMNP-based aggregation assays, the fMNPs serve as the detection elements. When immersed in protein rich media, off-target proteins tend to non-specifically adsorb to fMNPs.^7–9^ The adsorbed layer of off-target proteins is commonly referred to as a protein corona.^10,11^ The protein corona can be made up of 2 layers: the irreversible tightly bound hard corona and the reversible loosely bound soft corona.^8,12–14^ The protein corona can mask targeting ligands on the fMNP surface, causing a decrease in fMNP targeting efficacy. Aggregation assay inaccuracies due to detection element-off-target protein interactions pose an obstacle to the clinical translation of fMNP-based aggregation assays. To this end, we have investigated the effects of off-target serum proteins, in the biological range, on target induced fMNP aggregation. Our aggregation assay is a model system that was developed in Moss *et al.*, 2024 and consists of biotinylated MNPs (biotin-PEG_4_-MNPs) that target streptavidin.^15^ Like Moss *et al.*, 2024, biotin-PEG_4_-MNP-streptavidin binding was transduced into a measurable signal with Lodestone Biomedical's Nanoparticle Characterization System (an AC magnetic spectrometer, NCS).^16^ Using the NCS, we monitored changes in biotin-PEG_4_-MNP 3rd harmonic phase (*ϕ*_3_) when in the presence of varying amounts of streptavidin. We refer to biotin-PEG_4_-MNP *ϕ*_3_ as a function of streptavidin concentration as a streptavidin-response curve.^15^ We compared the resolution and dynamic range (measurable concentration range^17^) of streptavidin-response curves prepared in 1× PBS and 50% fetal bovine serum (FBS). 50% FBS has serum protein concentrations similar to those found *in vivo*. Therefore, the 50% FBS streptavidin-response curve demonstrated the effects of off-target serum proteins, in the biological range, on target induced fMNP aggregation.

It has been widely reported in the literature that protein corona formation can significantly decrease functionalized nanoparticle (fNP) targeting efficacy.^10,18–21^ For example, silica NPs engineered to sense an azide functionalized substrate, underwent significant decreases in efficacy after exposure to protein rich media.^22^ Mirshafiee *et al.* 2013 conjugated bicyclononyne (BCN) to 75 nm fluorescent silica NPs. The authors prepared 3 types of BCN-silica NPs: those incubated in (1) 10% serum, (2) 100% serum, and those kept (3) pristine. The pristine BCN-silica NPs showed significant binding to the azide substrate. Azide substrate binding decreased by 94% and 99% for the BCN-silica NPs incubated in 10% and 100% serum solutions, respectively.^22^ Their results demonstrate a decrease in BCN-silica NP efficacy due to interactions with off-target serum proteins.^22^ The extent of protein corona formation may vary with NP size, shape,^23^ and surface decoration,^18,24,25^ as well as environmental factors including diluent pH,^26^ electrolyte concentration,^27^ and temperature.^7^ Off-target serum protein-NP adsorption has also been leveraged to impart therapeutically advantageous characteristics to NPs, including enhanced stability^14,28–31^ and antifouling properties.^19,32^ Wiogo *et al.*, 2011, Lim *et al.*, 2009, and Aires *et al.*, 2015 have demonstrated increases in MNP stability when suspended in FBS solutions.^14,28–30^ Wiogo *et al.*, 2011 found that MNPs suspended in RPMI-1640 solutions containing 4% to 10% (v/v) FBS were more stable than MNPs suspended in RPMI-1640 solutions that were void of serum proteins.^14^ RPMI-1640 is a cell culture medium. The MNPs underwent a ∼1 µm increase in hydrodynamic diameter (*D*_Hyd_) after a ∼ 2-hour incubation in an RPMI-1640 solution that was void of serum proteins. However, MNP *D*_Hyd_ remained stable for 16 hours in RPMI-1640 solutions containing 4% to 10% (v/v) FBS. Conversely, Rabel *et al.*, 2021 observed a decrease in iron oxide particle stability due to interactions with serum proteins in human plasma solutions.^33^ They observed large agglomerates of poly(ethyleneimine) coated magnetite and silica coated maghemite particles while suspended in human plasma solutions.^33^

There are also studies that demonstrate the impact of protein corona formation on the utility of MNPs as negative contrast agents for magnetic resonance imaging (MRI).^8,9,30^ Wherein the relationship between protein corona formation and MNP transverse relaxivity are highlighted. The impact of off target serum proteins on fMNP-based biosensing was previously investigated in Zhang *et al.*, 2013. The authors compared ssDNA detection in PBS *versus* serum.^2^ A 100 pM ssDNA detection limit was observed in PBS, while a 400 pM detection limit was observed in serum.^2^ Therefore, the authors observed a decrease in fMNP target sensitivity in serum, compared to PBS. The detection limit was determined *via* investigating changes in the ratio of the amplitudes of the 5th harmonic (*A*_5_) to the 3rd harmonic (*A*_3_). Our work differs from that of Zhang *et al.*, 2013 in that we are studying changes in biotin-PEG_4_-MNP biosensor resolution and dynamic range, *via* investigating changes in biotin-PEG_4_-MNP *ϕ*_3_ as a function of target concentration, instead of the limit of detection *via* investigating changes in the ratio of *A*_5_ to *A*_3_. Further experimental demonstration of the impact of protein corona on MPS-based bioassays is necessary given that protein corona formation may increase fMNP stability (leading to a decrease in sensitivity) and mask their targeting ligands (leading to an increase in sensitivity). Since protein corona formation tends to increases fMNP stability, one may expect the biotin-PEG_4_-MNPs to experience decreased streptavidin sensitivity while suspended in 50% FBS, compared to 1× PBS. Conversely, given that a decrease in fMNP ligand density increases their target sensitivity^15,34^ (wherein Takae *et al.*, 2005 demonstrated a decrease in gold (Au)NP surface area ligand coverage led to an increase in assay sensitivity^34^) and protein corona formation may mask a portion of the available biotin on the biotin-PEG_4_-MNP surface, one may expect the biotin-PEG_4_-MNPs to experience an increase in streptavidin sensitivity while suspended in 50% FBS, compared to 1× PBS.

In this work, we demonstrate the impact of protein corona on fMNP-based biosensing. This study can aid in the development of wash-free fNP-based aggregation assays. Wash-free fNP-based aggregation assays may be suited for *in vivo* use, assuming the NPs have a biocompatible core (such as gold, silver, or magnetite). Specifically, fMNPs have the potential to enable more personalized medicine *via in vivo* biomarker monitoring. The biologic environment will contain various off-target proteins that may adsorb to the fMNPs. Therefore, understanding the effects of protein corona on fMNP target sensitivity can further progress the technology toward *in vivo* clinical use.

## 2 Materials and methods

### 2.1 Materials

Amine functionalized 25 nm magnetite (SHA 25) particles were purchased from Ocean Nanotech, San Diego, CA, USA. *N*-hydroxysuccinide (NHS)-PEG_4_-biotin and FBS were purchased from Thermo Fisher Scientific, Waltham, MA, USA. NHS-PEG_4_-methoxy was purchased from BroadPharm, San Diego, CA, USA. Streptavidin was purchased from Rockland Immunochemicals, Pottstown, PA, USA. Monomeric streptavidin (mono-streptavidin) was purchased from Millipore Sigma, Burlington, MA, USA.

### 2.2 Conjugating NHS-PEG_4_-ligands to SHA 25 MNPs

Two Eppendorf tubes containing 300 µL of 5 mg Fe per mL SHA 25 MNP solutions were centrifuged at 13 500 RCF for 30 minutes at 20 °C. Once the centrifugation was complete, the diluent was aspirated and the SHA 25 MNPs were resuspended in 300 µL of deionized water. Quantities of 2 mg of NHS-PEG_4_-biotin are stored in each vial of the Thermo Scientific No-WeighTM Format. To prevent moisture condensation, we allowed the NHS-PEG_4_-biotin and NHS-PEG_4_-methoxy to equilibrate to room temperature before opening each vial. To produce biotin-PEG_4_-SHA 25 MNPs with ∼10 available biotins for streptavidin binding per MNP, the NHS-PEG_4_-biotin was diluted to 0.50 mg mL^−1^ with deionized water, then 1.54 µL of the 0.50 mg mL^−1^ NHS-PEG_4_-biotin solution was added to one of the Eppendorf tubes containing the SHA 25 MNP solutions. To produce methoxy-PEG_4_-SHA 25 MNPs with ∼10 PEG_4_-methoxy ligands per MNP, 100 mg of NHS-PEG_4_-methoxy was diluted to 20 mg mL^−1^ with dimethyl sulfoxide and further diluted to 0.50 mg mL^−1^ with deionized water. Then 0.88 µL of the 0.50 mg mL^−1^ NHS-PEG_4_-methoxy solution was added to the 2nd Eppendorf tube containing an SHA 25 MNP solution. Next, the mixtures were left to incubate at room temperature for 45 minutes. Once the incubation was complete, each mixture was centrifuged at 13 500 RCF for 45 minutes at 20 °C eight times. After each centrifugation, the diluent was aspirated and the biotin-PEG_4_-MNP and methoxy-PEG_4_-MNP solutions were resuspended in 300 µL of deionized water. The *A*_3_ of SHA 25 MNP serial dilutions (0, 1, 2, 3, 4 and 5 g L^−1^ Fe concentrations) were measured with the NCS. The *A*_3_ of the biotin-PEG_4_-MNP and methoxy-PEG_4_-MNP solutions were compared with those of the SHA 25 MNP serial dilutions to determine the Fe concentration of the biotin-PEG_4_-MNP and methoxy-PEG_4_-MNP solutions. Lastly, the biotin-PEG_4_-MNP and methoxy-PEG_4_-MNP solutions were diluted to 1 mg Fe per mL based on their *A*_3_.

### 2.3 Magnetic particle spectroscopy measurements

Streptavidin-response curves were prepared in 1× PBS, 50% FBS, 1 µM bovine serum albumin (BSA), and 1 µM mono-streptavidin solutions. Wherein we prepared streptavidin serial dilutions spanning from 0.0 nM to 20.9 nM streptavidin in 1× PBS and 50% FBS for biotin-PEG_4_-MNPs and methoxy-PEG_4_-MNPs. We also prepared an additional 1× PBS streptavidin-response curve spanning from 0.0 nM to 145 nM streptavidin in 1× PBS for biotin-PEG_4_-MNPs and methoxy-PEG_4_-MNPs. As well as 1 µM BSA and 1 µM mono-streptavidin streptavidin-response curves spanning from 0.0 nM to 145 nM streptavidin in 1 µM BSA and 1 µM mono-streptavidin for biotin-PEG_4_-MNPs. An additional experiment was conducted to determine the impact of a decreased ligand density due to protein corona masking on streptavidin induced biotin-PEG_4_-MNP aggregation, wherein biotin-PEG_4_-MNPs underwent a ∼12-hour room temperature incubation in a 1 µM mono streptavidin solution (3 mono streptavidins per MNP). Next, the biotin-PEG_4_-MNPs were exposed to a streptavidin serial dilution spanning from 0 to 145 nM streptavidin in 1× PBS.

Each MPS sample contained 2.5 µL of a 1 mg Fe per mL biotin-PEG_4_-MNP or methoxy-PEG_4_-MNP solution mixed with 2.0 µL of a streptavidin serial dilution sample. Each MPS sample was prepared in triplicate. The *ϕ*_3_ of each sample was measured in triplicate with the NCS after an ∼40-minute incubation at room temperature. At the onset of each measurement, the NCS identifies the magnetic center of each sample. Next, the sample *ϕ*_3_ is measured at 7 points along the *z*-axis of the sample (from bottom to top). Where the center point is located at the magnetic center of the sample. The 7 point *ϕ*_3_ measurement is repeated 4 times during each NCS measurement. Once the streptavidin-response curves were complete, we identified the streptavidin concentration at which the maximum *ϕ*_3_ occurred in 1× PBS and 50% FBS. The maximum *ϕ*_3_ occurred at 97 nM streptavidin and 18 nM streptavidin for 1× PBS and 50% FBS, respectively. 3 additional MPS samples containing 97 nM streptavidin 1× PBS plus 3 more MPS samples containing 18 nM streptavidin 50% FBS were prepared and stored for further DLS analysis.

We compared the resolution and dynamic range of the 1× PBS and 50% FBS biotin-PEG_4_-MNP streptavidin-response curves. These data were meant to demonstrate the effects of off-target serum proteins on streptavidin induced biotin-PEG_4_-MNP aggregation. First, we identified the aggregation assay dynamic range while operating in each diluent. Then, we used a 3 step statistical framework to determine whether the data in the dynamic range possessed statistically significant variations. The smallest Δ streptavidin concentration for which the *ϕ*_3_ data were consistently statistically significantly different was identified as the aggregation assay's resolution for a given diluent. Once the dynamic range for a given diluent was identified, we performed 2-sided *t*-tests on the data to determine whether they possessed statistically significant variations in *ϕ*_3_. Before preforming the *t*-test, we implemented a Barlett Test (“vartestn” Matlab command) with a 95% threshold to determine whether the data within the dynamic range came from normal distributions with equal but unknown variances. This result was meant to inform our *t*-test parameters wherein we used either the (…,“Vartype” “equal”) or (…,“Vartype” “unequal”) command if the null hypothesis of the Barlett test was rejected or if there was a failure to reject the null hypothesis, respectively. Then, we adjusted the *t*-test threshold to reduce the likelihood of false positive errors due to multiple comparisons, *via* Bonferonni correction. Lastly, *t*-tests with appropriate variance and threshold parameters were conducted on the data within the dynamic region of each streptavidin-response curve.

It is important to note that different batches of SHA 25 MNPs were used throughout the manuscript, which led to variation in Δ*ϕ*_3_ and a small variation in the streptavidin concentration at which the maximum *ϕ*_3_ occurred for biotin-PEG_4_-MNPs preparaed using identical methods.

### 2.4 Physical characterization of biotin-PEG_4_-MNPs

Biotin-PEG_4_-MNP aliquots (80 mg Fe per L concentration) were submitted to DLS, to verify the time evolution of biotin-PEG_4_-MNP *D*_Hyd_ in 1× PBS and 50% FBS at room temperature for 8 hours, with *D*_Hyd_ measurements every 20 minutes. Each DLS sample was prepared in duplicate. The DLS measurements were conducted with a Zetasizer Ultra Red (Malvern Panalytical). 70 µL of each 80 mg Fe per L biotin-PEG_4_-MNP solution were transferred to disposable micro cuvettes (ZEN0040). The measurements were conducted at 25 °C with a light scattering collection angle of 174.7° (back scatter). We also conducted *D*_Hyd_ measurements for biotin-PEG_4_-MNPs in 0% (1× PBS), 10%, 20%, 30%, 40%, and 50% FBS solutions to gauge the extent of protein corona formation with increasing diluent serum protein concentration. These *D*_Hyd_ measurements were conducted in quintuplicate. The temperature and light scattering collection angle were identical to those used to monitor the time evolution of biotin-PEG_4_-MNP *D*_Hyd_. Next, biotin-PEG_4_-MNP aliquots (10 mg Fe per L concentration) were submitted to DLS, to verify their zeta potential in 1× PBS and 50% FBS. 1 mL of each 10 mg Fe per L biotin-PEG_4_-MNP solution was injected into a disposable folded capillary cell (DTS1070). Our zeta potential measurements were conducted in quintuplicate, with *D*_Hyd_ measurements before and after each zeta potential measurement to monitor biotin-PEG_4_-MNP stability.

The 3 MPS samples prepared in Section 2.3, containing 97 nM streptavidin 1× PBS and 18 nM streptavidin 50% FBS solutions, were further diluted for DLS measurement. 11.4 µL of each MPS sample solution was added to 68.6 µL of 1× PBS, to prepare 80 mg Fe per L solutions. Then, 70 µL of each 80 mg Fe per L biotin-PEG_4_-MNP solution were transferred to disposable micro cuvettes (ZEN0040) to compare the aggregate *D*_Hyd_ for fully clustered biotin-PEG_4_-MNPs in 1× PBS *versus* 50% FBS. These *D*_Hyd_ measurements were conducted in quintuplicate. The temperature and light scattering collection angle were identical to those used to monitor the time evolution of biotin-PEG_4_-MNP *D*_Hyd_.

We conducted statistical analyses on the data gauging biotin-PEG_4_-MNP stability while suspended in 1× PBS or 50% FBS for an 8-hour room temperature incubation, the data gauging the extent of protein corona formation with increasing diluent serum protein concentrations as well as the data comparing biotin-PEG_4_-MNP aggregate *D*_Hyd_ for solutions containing 97 nM streptavidin 1× PBS *versus* 18 nM streptavidin 50% FBS solutions. Concerning the data gauging the extent of protein corona formation with increasing diluent serum protein concentrations, we conducted *t*-tests on the data to determine whether there were statistically significant variations in biotin-PEG_4_-MNP *D*_Hyd_ for 0% (1× PBS), 10%, 20%, 30%, 40%, and 50% FBS solutions. Similar to the statistical analysis procedure implemented in Section 2.3, we (1) performed a Barlett Test on the data, (2) adjusted the threshold to account for multiple comparisons *via* Bonferonni correction, (3) and lastly we conducted *t*-tests on the data with the appropriate variance and threshold parameters. Concerning the data gauging biotin-PEG_4_-MNP stability while suspended in 1× PBS or 50% FBS for an 8-hour room temperature incubation, we used the 3-step statistical framework on the time dependent biotin-PEG_4_-MNP *D*_Hyd_ data to determine whether biotin-PEG_4_-MNP *D*_Hyd_ statistically significantly differed throughout the 8-hour room temperature incubation in either 1× PBS or 50% FBS. A Barlett Test and *t*-test were also applied to the data comparing biotin-PEG_4_-MNP aggregate *D*_Hyd_ for solutions containing 97 nM streptavidin 1× PBS *versus* 18 nM streptavidin 50% FBS solutions to determine whether there were statistically significant variations in biotin-PEG_4_-MNP aggregate *D*_Hyd_.

## 3 Results

### 3.1 Impact of off-target serum proteins on streptavidin induced biotin-PEG_4_-MNP aggregation

**Fig. 1:**
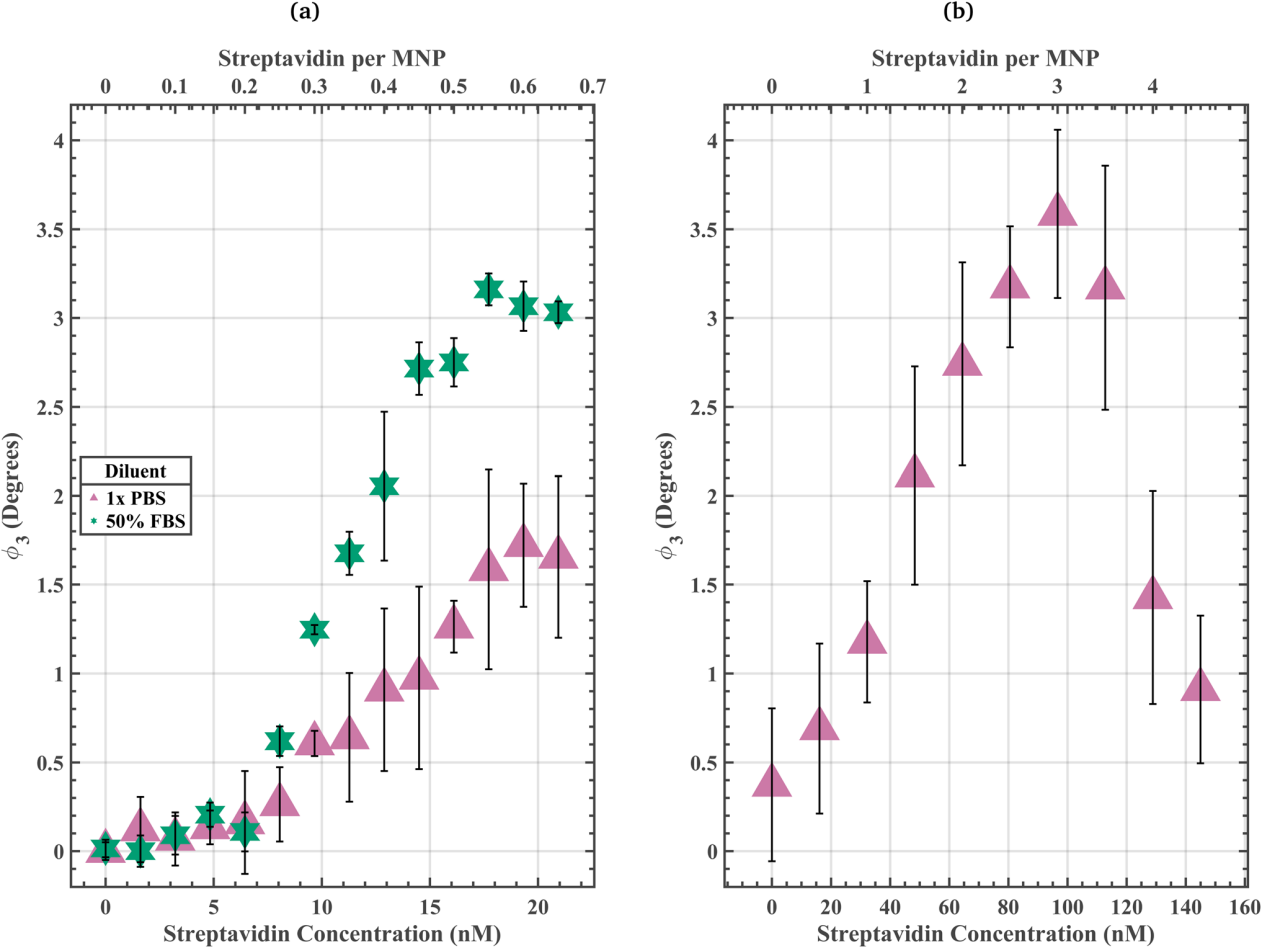
(a) Biotin-PEG_4_-MNP *ϕ*_3_ as a function of streptavidin concentrations (streptavidin-response curve) spanning from 0 to 20.94 nM streptavidin in 1× PBS and 50% FBS. Where biotin-*PEG*_4_-MNP *ϕ*_3_ in a 1.61 nM streptavidin 50% FBS solution and 0 nM streptavidin 1× PBS solution were regarded as 0° for the 50% FBS and 1× PBS streptavidin-response curves, respectively. The biotin-PEG_4_-MNPs did not fully aggregate in 1× PBS solutions containing ≤20.94 nM streptavidin. Conversely, the biotin-PEG_4_-MNPs fully aggregated in a 50% FBS solution containing 18 nM streptavidin. (b) Biotin-PEG_4_-MNP streptavidin-response curve spanning from 0 to 144.97 nM streptavidin in 1× PBS. The biotin-PEG_4_-MNPs fully aggregated in a 1× PBS solution containing 97 nM streptavidin. These data suggest that the off-target serum proteins present in 50% FBS increased biotin-PEG_4_-MNP streptavidin sensitivity. The error bars represent a 95% confidence interval on each mean value.

The 50% FBS streptavidin-response curve had a resolution of 3.22 nM streptavidin and a dynamic region spanning from 4.83 nM to 14.50 nM streptavidin (Fig. 1a). It is important to note that the aggregation assay's resolution corresponds to the Δ streptavidin concentration for which the *ϕ*_3_ data were consistently statistically significantly different within the dynamic range for a given diluent. The resolution does not constrain the set of possible input target concentrations. For 4.83 nM, 6.44 nM, 8.05 nM, 9.66 nM, 11.28 nM, 12.89 nM, and 14.50 nM streptavidin 50% FBS solutions, the biotin-PEG_4_-MNPs had *ϕ*_3_ of 0.21 ± 0.07°, 0.11 ± 0.11°, 0.62 ± 0.08°, 1.25 ± 0.03°, 1.68 ± 0.12°, 2.05 ± 0.42°, and 2.72 ± 0.15°, respectively. Where biotin-PEG_4_-MNP *ϕ*_3_ in a 1.61 nM streptavidin 50% FBS solution was regarded as 0°. Only samples with a difference in streptavidin concentration ≥3.22 nM streptavidin had statistically significant variations in *ϕ*_3_ (threshold 99.17%). Fig. 1a also contains biotin-PEG_4_-MNP *ϕ*_3_ data for 4.83 nM, 6.44 nM, 8.05 nM, 9.66 nM, 11.28 nM, 12.89 nM, and 14.50 nM streptavidin 1× PBS solutions (void of off-target serum proteins). For 4.83 nM, 6.44 nM, 8.05 nM, 9.66 nM, 11.28 nM, 12.89 nM, and 14.50 nM streptavidin 1× PBS solutions, the biotin-PEG_4_-MNPs had *ϕ*_3_ of 0.13 ± 0.10°, 0.16 ± 0.29°, 0.26 ± 0.21°, 0.61 ± 0.07°, 0.64 ± 0.36°, 0.91 ± 0.46°, and 0.98 ± 0.51° respectively. Where biotin-PEG_4_-MNP *ϕ*_3_ in a 0 nM streptavidin 1× PBS was regarded as 0°. There were no statistically significant variations (99.17% threshold) amongst these data. These data are meant to act as calibration curves, that relate MNP *ϕ*_3_ to the target concentration within a sample solution. If the data within the dynamic region are not statistically significantly different, it is useless for target biomolecule detection. Therefore, the 50% FBS streptavidin-response curve could have been used for target biomolecule detection, whereas the 1× PBS streptavidin-response curve could not have been used for target biomolecule detection. These data suggest that the presence of the off-target serum proteins present in 50% FBS increased the aggregation assay target sensitivity and resolution, enabling it to quantify lower streptavidin concentrations.

**Fig. 2:**
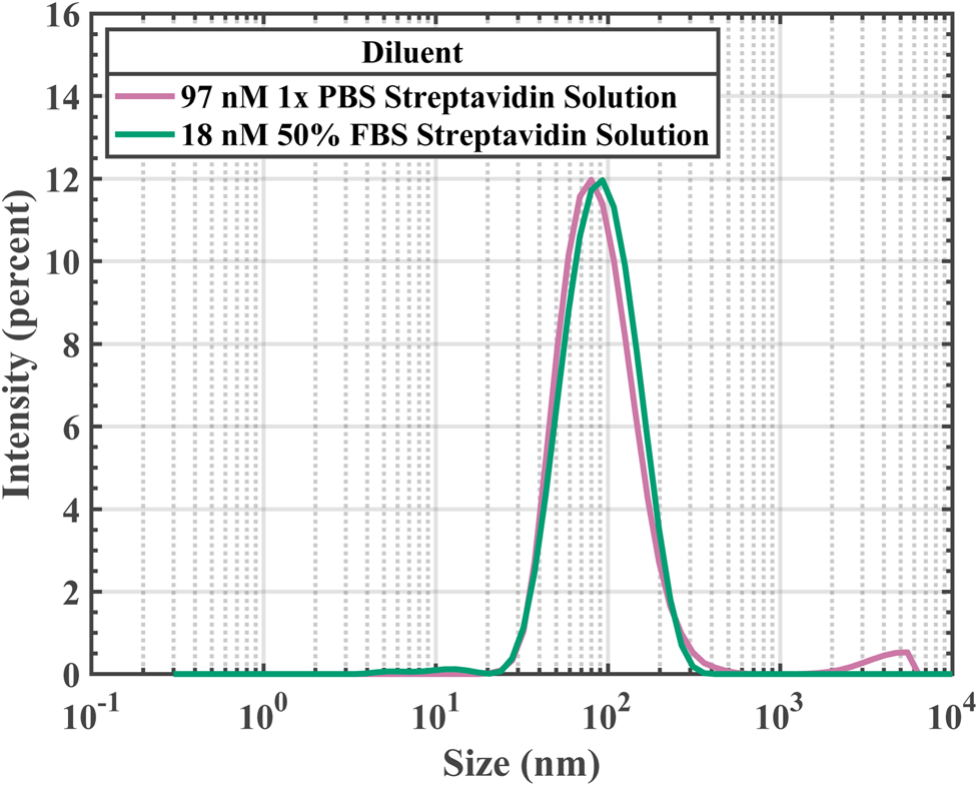
Biotin-PEG_4_-MNP *D*_Hyd_ while suspended in a 97 nM 1× PBS streptavidin solution was indistinguishable from biotin-PEG_4_-MNP *D*_Hyd_ while suspended in a 18 nM 50% FBS streptavidin solution. These data suggest that the off-target serum proteins present in 50% FBS increased biotin-PEG_4_-MNP streptavidin sensitivity.

A 1× PBS streptavidin-response curve spanning higher target concentrations yielded meaningful data capable of target biomolecule detection (Fig. 1b). This 1× PBS streptavidin-response curve had a resolution of 64.43 nM streptavidin and a dynamic region spanning from 0 nM to 96.65 nM streptavidin. For 0 nM, 16.11 nM, 32.22 nM, 48.32 nM, 64.43 nM, 80.54 nM, and 96.65 nM streptavidin 1× PBS solutions, the biotin-PEG_4_-MNPs had *ϕ*_3_ of 0.37 ± 0.43°, 0.69 ± 0.48°, 1.18 ± 0.34°, 2.11 ± 0.61°, 2.74 ± 0.57°, 3.18 ± 0.34°, and 3.59 ± 0.47°, respectively. Where biotin-PEG_4_-MNP *ϕ*_3_ in a 0 nM streptavidin 1× PBS solution was regarded as 0°. Only samples with a difference in streptavidin concentration ≥64.43 nM streptavidin had statistically significant variations in *ϕ*_3_ (threshold 99.76%). Our aggregation assay had a finer resolution in 50% FBS solutions (3.22 nM streptavidin), compared to in 1× PBS solutions (64.43 nM streptavidin). We also identified the streptavidin concentration at which the maximum *ϕ*_3_ occurred for biotin-PEG_4_-MNPs suspended in 1× PBS and 50% FBS streptavidin solutions. Biotin-PEG_4_-MNPs suspended in 50% FBS achieved a maximum *ϕ*_3_ at a lower streptavidin concentration compared to biotin-PEG_4_-MNPs suspended in 1× PBS. Biotin-PEG_4_-MNPs suspended in 1× PBS and 50% FBS solutions had maximum *ϕ*_3_ in solutions containing 97 nM streptavidin and 18 nM streptavidin, respectively. Moreover, biotin-PEG_4_-MNPs suspended in 97 nM streptavidin 1× PBS and 18 nM streptavidin 50% FBS solutions had *ϕ*_3_ of 3.80 ± 0.47° and 3.16 ± 0.09°, respectively. The maximum *ϕ*_3_ for biotin-PEG_4_-MNPs in 1× PBS did not statistically significantly differ (95% threshold) from the maximum *ϕ*_3_ for biotin-PEG_4_-MNPs in 50% FBS. Additionally, we measured the *D*_Hyd_ of biotin-PEG_4_-MNP aggregates present in the 97 nM streptavidin 1× PBS and 18 nM streptavidin 50% FBS solutions (Fig. 2 and Table 1). The aggregates belonging to the 97 nM streptavidin 1× PBS and 18 nM streptavidin 50% FBS solutions had *D*_Hyds_ of 97 ± 13 nm and 100 ± 4 nm, respectively. The *D*_Hyd_ for biotin-PEG_4_-MNP aggregates belonging to the 97 nM streptavidin 1× PBS solution did not statistically significantly differ from the *D*_Hyd_ for biotin-PEG_4_-MNP aggregates belonging to the 18 nM streptavidin 50% FBS solution. These data further demonstrate an increase in aggregation assay target sensitivity in 50% FBS, when compared to aggregation assay target sensitivity in 1× PBS.

**Table 1:** *D*_Hyd_ and zeta potential data for biotin-PEG_4_-MNPs suspended in 1× PBS and 50% FBS. As well as *D*_Hyd_ data for biotin-PEG_4_-MNPs suspended in 97 nM streptavidin 1× PBS and 18 nM streptavidin 50% FBS solutions. The reported errors represent a 95% confidence interval on each mean value

Biotin-PEG_4_-MNP diluent	Hydrodynamic diameter (nm)	Zeta potential (mV)
0 nM streptavidin in 1× PBS	62 ± 1	−1.2 ± 13.6
0 nM streptavidin in 50% FBS	63 ± 5	−8.3 ± 5.3
97 nM streptavidin in 1× PBS	97 ± 13	—
18 nM streptavidin in 50% FBS	100 ± 4	—

**Fig. 3:**
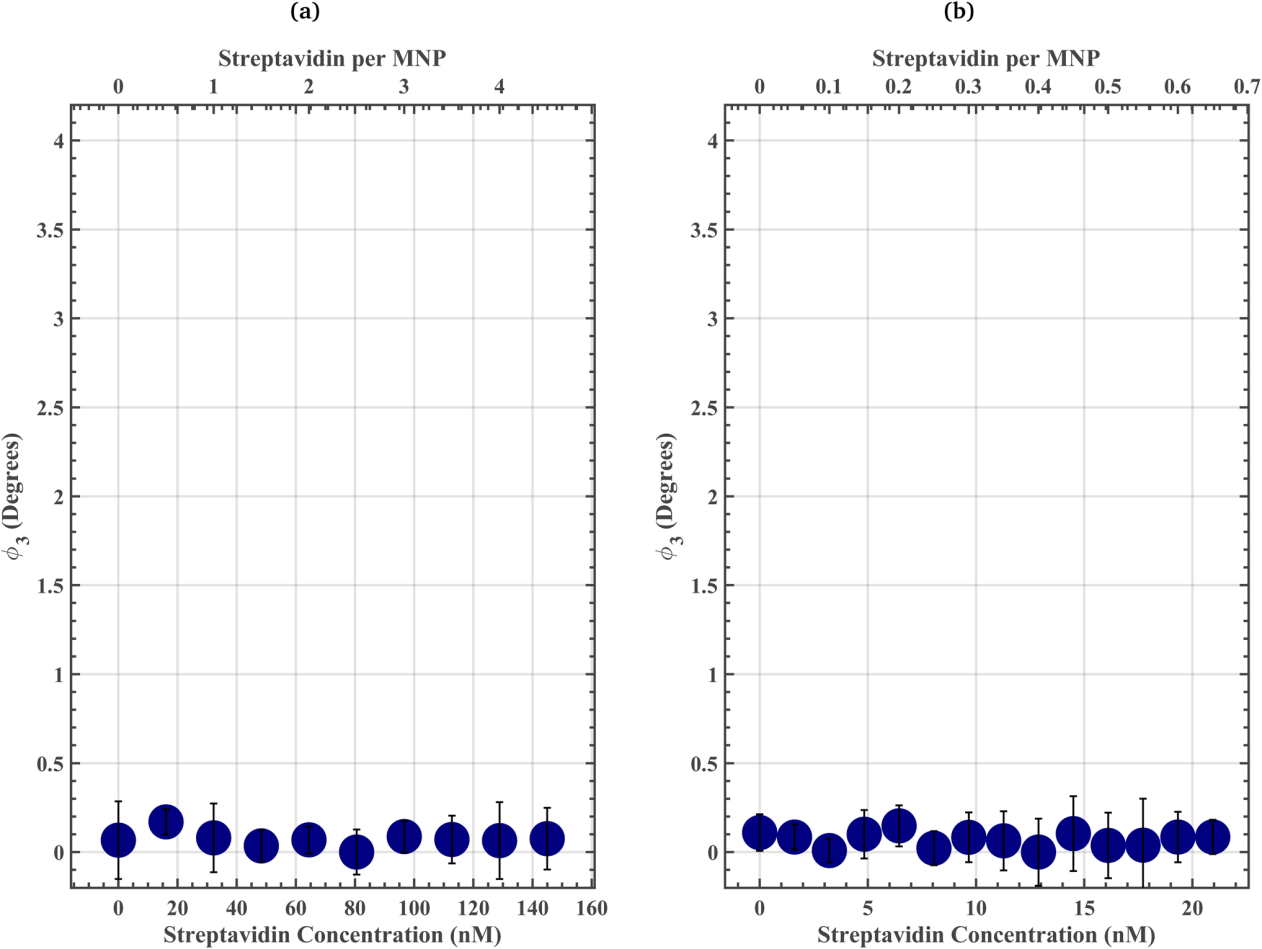
(a) Methoxy-PEG_4_-MNP streptavidin-response curves spanning from 0 to 144.97 nM streptavidin in 1× PBS. Where methoxy-PEG_4_-MNP *ϕ*_3_ in an 80.54 nM streptavidin 1× PBS solution was regarded as 0°. These data suggest that non-specific streptavidin binding did not cause statistically significant variations in *ϕ*_3_ (threshold 99.89%). (b) Methoxy-PEG_4_-MNP streptavidin-response curve spanning from 0 to 20.94 nM streptavidin in 50% FBS. Where methoxy-PEG_4_-MNP *ϕ*_3_ in an 12.89 nM streptavidin 50% FBS solution was regarded as 0°. There were no statistically significant variations in *ϕ*_3_ (threshold 99.99%). These data suggest that the presence of off-target proteins did not increase non-specific streptavidin binding, evident by the lack of statistically significant variations in *ϕ*_3_ for methoxy-PEG_4_-MNPs suspended in 50% FBS. Therefore, non-specific streptavidin binding did not contribute to the change in assay resolution from 64.43 nM streptavidin in 1× PBS (in the absence of off-target proteins) to 3.22 nM streptavidin in 50% FBS (in the presence of off-target proteins). The error bars represent a 95% confidence interval on each mean value.

We also prepared methoxy-PEG_4_-MNP streptavidin-response curves in 1× PBS and 50% FBS to disentangle the role of the protein corona from that of surface functionalization (Fig. 3). The methoxy-PEG_4_-MNP streptavidin-response curve in 1× PBS spanning from 0 to 144.97 nM streptavidin suggests that non-specific streptavidin binding did not cause statistically significant variations in *ϕ*_3_ (threshold 99.89%). The methoxy-PEG_4_-MNP streptavidin-response curve in 50% FBS spanning from 0 to 20.94 nM streptavidin suggest that the presence of off-target proteins did not increase non-specific streptavidin binding, evident by the lack of statistically significant variations in *ϕ*_3_ for methoxy-PEG_4_-MNPs suspended in 50% FBS. Therefore, non-specific streptavidin binding did not contribute to the change in assay resolution from 64.43 nM streptavidin in 1× PBS (in the absence of off-target proteins) to 3.22 nM streptavidin in 50% FBS (in the presence of off-target proteins) (Fig. 3).

### 3.2 Impact of decreased ligand density (due to protein corona masking) on streptavidin induced biotin-PEG_4_-MNP aggregation

**Fig. 4:**
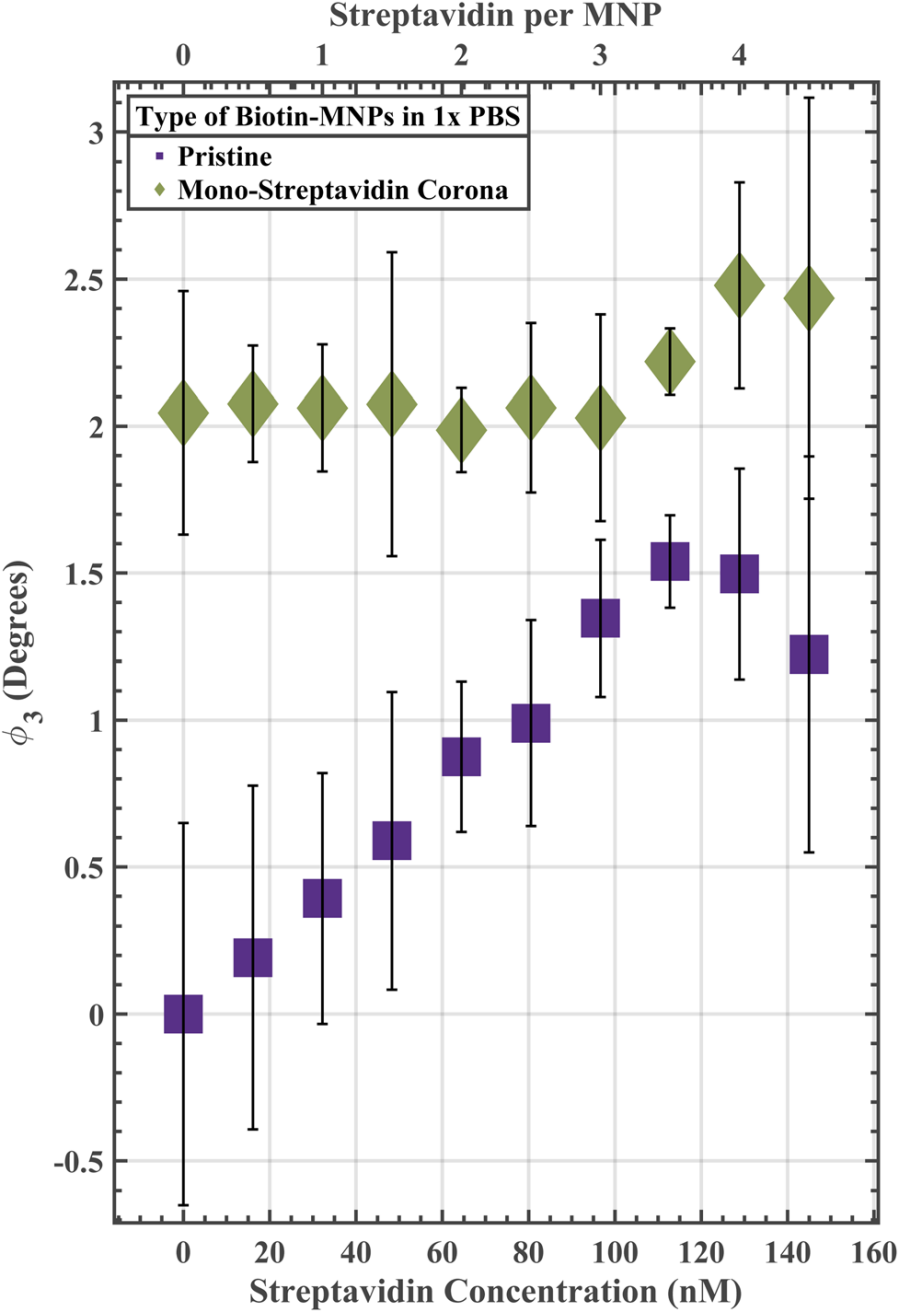
Pristine biotin-PEG_4_-MNP and mono-streptavidin corona biotin-PEG_4_-MNP streptavidin-response curves spanning from 0 to 144.97 nM streptavidin in 1× PBS. Where biotin-PEG_4_-MNP *ϕ*_3_ in a 0 nM streptavidin 1× PBS solution was regarded as 0°. A decreased ligand density due to a mono-streptavidin corona masking a portion of the available biotin for streptavidin binding decreased Δ*ϕ*_3_ and shifted the streptavidin concentration at which the maxiumum *ϕ*_3_ occurred to a higher streptavidin concentration. These phenomena were not observed for biotin-PEG_4_-MNPs targeting streptavidin in 50% FBS. These data suggest that a decrease in ligand density due to protein corona masking did not contribute to the change in assay resolution from 64.43 nM streptavidin in 1× PBS (in the absence of off-target proteins) to 3.22 nM streptavidin in 50% FBS (in the presence of off-target proteins). The error bars represent a 95% confidence interval on each mean value.

Biotin-PEG_4_-MNPs underwent an approximate 12-hour room temperature incubation in a 1 µM mono streptavidin solution (3 mono-streptavidins per MNP). Next, the biotin-PEG_4_-MNPs were exposed to a streptavidin serial dilution spanning from 0 to 144.97 nM streptavidin in 1× PBS (Fig. 4). The bound mono-streptavidin was meant to simulate a protein corona that masked a portion of available biotin for streptavidin binding on the MNP surface. If the mono-streptavidin corona impacts biotin-PEG_4_-MNP streptavidin sensitivity in a similar manner as the off-target proteins present in 50% FBS, we can conclude that a decrease in available biotin for streptavidin binding due to protein corona masking strongly contributed to the change in assay resolution from 64.43 nM streptavidin in 1× PBS (in the absence of off-target proteins) to 3.22 nM streptavidin in 50% FBS (in the presence of off-target proteins). Conversely, if the mono-streptavidin corona impacts biotin-PEG_4_-MNP streptavidin sensitivity in a dissimilar manner than the off-target proteins present in 50% FBS, we can conclude that an alternative unknown mechanism strongly contributed to the diluent dependent changes in assay resolution. Pristine biotin-PEG_4_-MNPs had a *ϕ*_3_ of 0 ± 0.65° in a 1× PBS solution void of streptavidin, while mono-streptavidin corona biotin-PEG_4_-MNPs had a *ϕ*_3_ of 2.05 ± 0.41° in a 1× PBS solution void of streptavidin. Therefore, the mono-streptavidin corona triggered a ∼2.05° increase in *ϕ*_3_. Where biotin-PEG_4_-MNP *ϕ*_3_ in a 0 nM streptavidin 1× PBS solution was regarded as zero. This *ϕ*_3_ increase may stem from corona induced *D*_Hyd_ increases and/or instability. The mono-streptavidin corona may have destabilized the biotin-PEG_4_-MNPs resulting in small non-target induced clusters. The mono-streptavidin corona biotin-PEG_4_-MNP streptavidin-response curve had a smaller Δ*ϕ*_3_ compared to the pristine biotin-PEG_4_-MNP streptavidin-response curve. Δ*ϕ*_3_ describes the change in *ϕ*_3_ between MNPs in solutions void of target and MNPs at the peak of target induced aggregation. The pristine biotin-PEG_4_-MNP streptavidin-response curve had a Δ*ϕ*_3_ of 1.54°, while the mono-streptavidin corona biotin-PEG_4_-MNP streptavidin-response curve had a Δ*ϕ*_3_ of 0.45°. We attribute the mono-streptavidin cororna induced decrease in Δ*ϕ*_3_ to a decrease in streptavidin sensitivity due to the presence of mono-streptavidin induced biotin-PEG_4_-MNP aggregates. Moreover, the streptavidin concentration at which the maxiumum *ϕ*_3_ occurred did not shift to a lower concentration as was observed for biotin-PEG_4_-MNPs suspended in 50% FBS. Pristine biotin-PEG_4_-MNPs and mono-streptavidin corona biotin-PEG_4_-MNPs had maximum *ϕ*_3_ in solutions containing 113 nM streptavidin and 129 nM streptavidin, respectively. Therefore, a decreased biotin density due to a mono-streptavidin corona masking a portion of the available biotin for streptavidin binding decreased Δ*ϕ*_3_ and shifted the streptavidin concentration at which the maximum *ϕ*_3_ occurred to a higher concentration of streptavidin. These phenomena were not observed for biotin-PEG_4_-MNPs targeting streptavidin in 50% FBS. These data demonstrate that the mono streptavidin corona impacts biotin-PEG_4_-MNP streptavidin sensitivity in a dissimilar manner than the off-target proteins present in 50% FBS. Which suggests that an alternative unknown mechanism strongly contributed to the diluent dependent changes in assay resolution.

### 3.3 Impact of a low affinity off-target protein population *versus* a high affinity off-target protein population on streptavidin induced biotin-PEG_4_-MNP aggregation

**Fig. 5:**
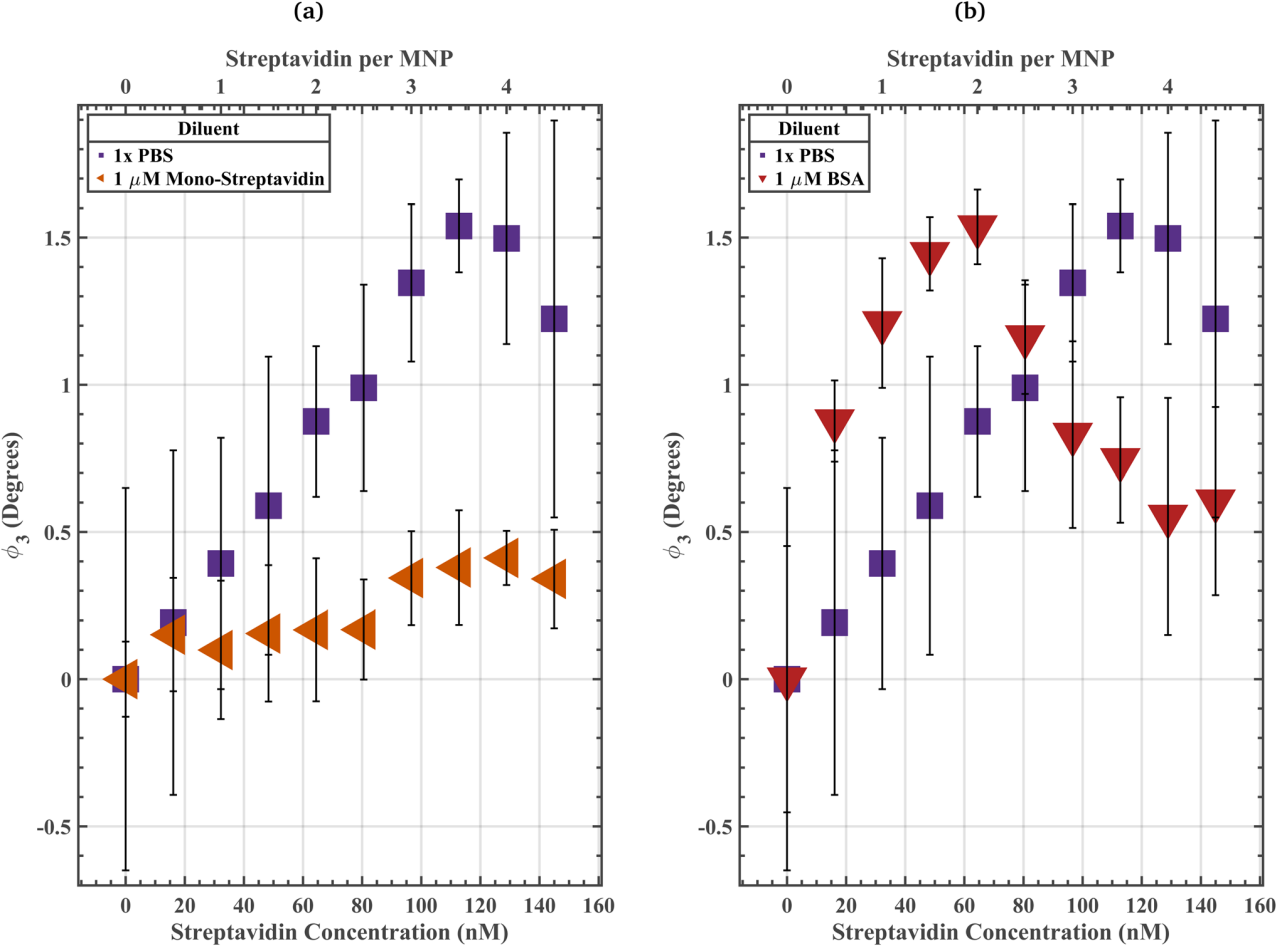
(a) Biotin-PEG_4_-MNP streptavidin-response curves spanning from 0 to 144.97 nM streptavidin in 1× PBS and 1 µM mono-streptavidin. Where biotin-PEG_4_-MNP *ϕ*_3_ in a 0 nM streptavidin 1× PBS solution and a 0 nM streptavidin 1 µM mono-streptavidin solution were regarded as 0° for the 1× PBS and 1 µM mono-streptavidin streptavidin-response curves, respectively. The biotin-PEG_4_-MNPs did not fully aggregate in 1 µM mono-streptavidin solutions with streptavidin concentrations spanning from 0 to 144.97 nM. Conversely, the biotin-PEG_4_-MNPs fully aggregated in a 1× PBS solution containing 113 nM streptavidin. These data suggest that a high affinity off-target protein population (mono-streptavidin) decreased biotin-PEG_4_-MNP streptavidin sensitivity. (b) Biotin-PEG_4_-MNP streptavidin-response curves spanning from 0 to 144.97 nM streptavidin in 1× PBS and 1 µM BSA. Where biotin-PEG_4_-MNP *ϕ*_3_ in a 0 nM streptavidin 1 µM BSA solution was regarded as 0° for the 1 µM BSA streptavidin-response curve. The biotin-PEG_4_-MNPs fully aggregated in a 1 µM BSA solution containing 64 nM streptavidin. These data suggest that a low affinity off-target protein population (BSA) increased biotin-PEG_4_-MNP streptavidin sensitivity. These data also suggest that competition with a low affinity off-target protein population strongly contributed to the change in assay resolution from 64.43 nM streptavidin in 1× PBS (in the absence of off-target proteins) to 3.22 nM streptavidin in 50% FBS (in the presence of off-target proteins). The error bars represent a 95% confidence interval on each mean value.

The streptavidin–biotin bond has a *k*_off_ of ∼10^−6^ s^−1^ (ref. 35) while the SavPhire mono-streptavidin–biotin bond has a *k*_off_ of ∼10^−5^ s^−1^. Wherein bond strength is inversely proportional to *k*_off_. We assume the BSA-biotin bond has a *k*_off_ several orders of magnitude larger than the *k*_off_ for mono-streptavidin since there is no specific interaction between BSA and biotin. The 1 µM mono streptavidin solution was meant to simulate the effects of a high affinity off-target protein population on streptavidin induced biotin-PEG_4_-MNP aggregation (Fig. 5a). The 1 µM BSA solution was meant to simulate the effects of low affinity off-target protein population on streptavidin induced biotin-PEG_4_-MNP aggregation (Fig. 5b). If either the low affinity off-target protein diluent solution (1 µM BSA solution) or high affinity off-target protein diluent solution (1 µM mono-streptavidin solution) impact biotin-PEG_4_-MNP streptavidin sensitivity in a similar manner as the off-target proteins present in 50% FBS, we can conclude that competition with either a low affinity off-target protein population or a high affinity off-target protein population strongly contributed to the change in assay resolution from 64.43 nM streptavidin in 1× PBS (in the absence of off-target proteins) to 3.22 nM streptavidin in 50% FBS (in the presence of off-target proteins). Biotin-PEG_4_-MNPs suspended in a 1 µM BSA solution achieved a maximum *ϕ*_3_ at a lower streptavidin concentration compared to biotin-PEG_4_-MNPs suspended in 1× PBS (Fig. 5b). The 1× PBS streptavidin-response curve and 1 µM BSA streptavidin-response curve had maximum *ϕ*_3_ in solutions containing 113 nM streptavidin and 64 nM streptavidin, respectively. Moreover, biotin-PEG_4_-MNPs suspended in 113 nM streptavidin 1× PBS and 64 nM streptavidin 1 µM BSA had *ϕ*_3_ of 1.64 ± 0.16° and 1.54 ± 0.13°, respectively. The maximum *ϕ*_3_ for biotin-PEG_4_-MNPs in 1× PBS did not statistically significantly differ (95% threshold) from the maximum *ϕ*_3_ for biotin-PEG_4_-MNPs in a 1 µM BSA solution. The biotin-PEG_4_-MNPs suspended in the high affinity off-target protein diluent (1 µM mono-streptavidin) did not undergo substantial levels of streptavidin induced aggregation (Fig. 5a). These data suggest that competition with a low affinity off-target protein population strongly contributed to the change in assay resolution from 64.43 nM streptavidin in 1× PBS (in the absence of off-target proteins) to 3.22 nM streptavidin in 50% FBS (in the presence of off-target proteins). While competition with a high affinity off-target protein population significantly decreased biotin-PEG_4_-MNP streptavidin sensitivity.

### 3.4 Impact of off-target serum proteins on biotin-PEG_4_-MNP *D*_Hyd_ and zeta potential

**Fig. 6:**
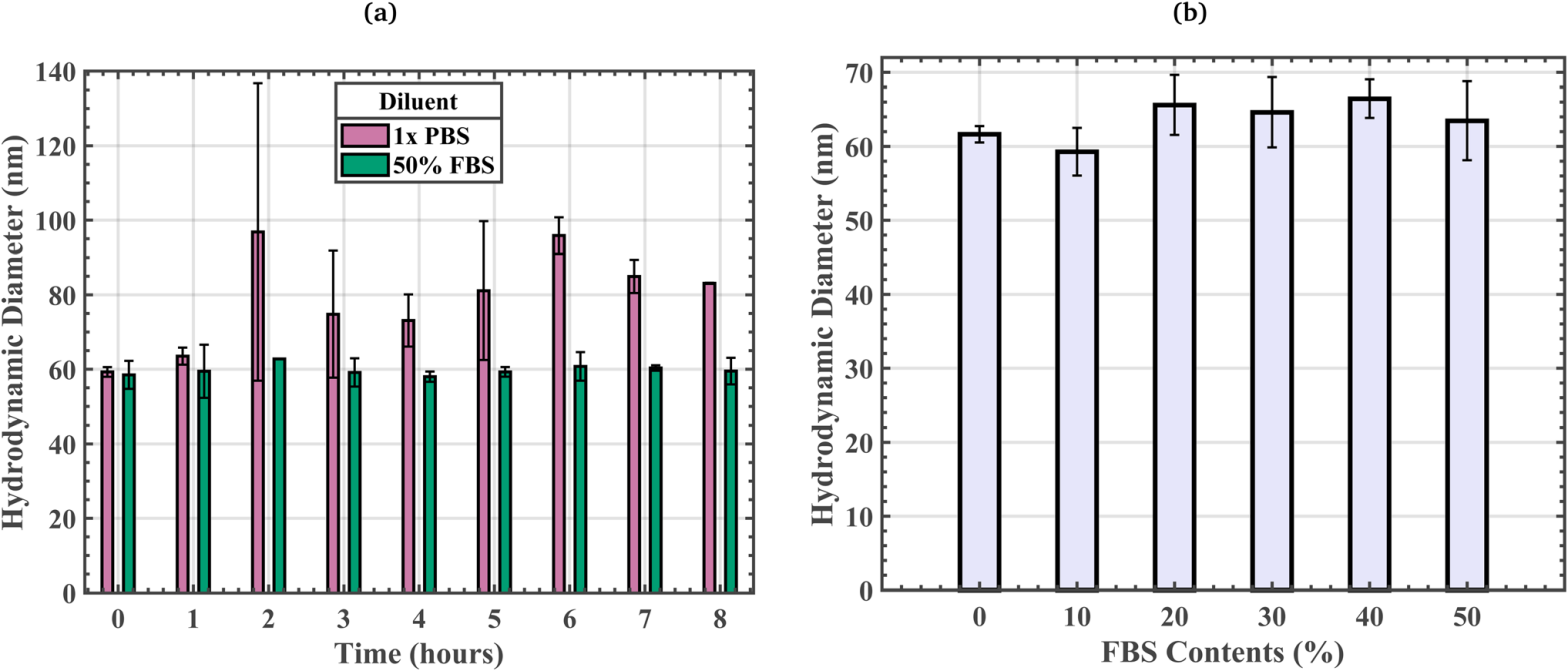
(a) Time evolution of biotin-PEG_4_-MNP *D*_Hyd_ while suspended in 1× PBS and 50% FBS at room temperature. These data suggest that the presence of the off-target serum proteins present in 50% FBS stabilized our biotin-PEG_4_-MNPs against aggregation for at least 8 hours. The error bars represent an 80% confidence interval on each mean value. (b) Biotin-PEG_4_-MNP *D*_Hyd_ in 0% (1× PBS), 10%, 20%, 30%, 40%, and 50% FBS solutions. The error bars represent a 95% confidence interval on each mean value.

We monitored the time evolution of biotin-PEG_4_-MNP *D*_Hyd_ while suspended in 1× PBS and 50% FBS at room temperature for 8 hours, with *D*_Hyd_ measurements every 20 minutes. Fig. 6 shows biotin-PEG_4_-MNP *D*_Hyd_ data at each hour of the incubation. The biotin-PEG_4_-MNPs suspended in 1× PBS underwent significant *D*_Hyd_ increases at hour 2 of the incubation. While the biotin-PEG_4_-MNPs suspended in 50% FBS did not undergo substantial *D*_Hyd_ fluctuations for the entirety of the 8-hour room temperature incubation. These data suggest that the presence of off-target serum proteins belonging to 50% FBS stabilized our biotin-PEG_4_-MNPs against aggregation for 8 hours at room temperature. Wiogo *et al.*, 2011, Lim *et al.*, 2009, and Aires *et al.*, 2015 similarly demonstrate an increase in MNP stability when suspended in solutions containing serum proteins.^14,28–30^ Fluctuations in biotin-PEG_4_-MNP *D*_Hyd_ while suspended in 1× PBS between hour 2 and hour 8 of the room temperature incubation were statistically insignificant (99.76% threshold). Moreover, one may be concerned that the 95% confidence intervals approach or exceed the *D*_Hyd_ values for fully aggregated biotin-PEG_4_-MNPs in 97 nM streptavidin 1× PBS and 18 nm streptavidin 50% FBS solutions (97 ± 1 nm and 97 ± 4 nm, respectively). This may suggest that biotin-PEG_4_-MNPs suspended in 1× PBS may undergo non-target induced aggregation after an approximate 2-hours room temperature incubation resulting in clusters comparable in size to target-induced aggregates. This could potentially lead to false positive errors during 1× PBS assay operation. However, SI Fig. S3a in Moss *et al.*, 2024 suggests that variations in biotin-PEG_4_-MNP *ϕ*_3_ throughout a 12-hour room temperature incubation were statistically insignificant (99.99% significance threshold).^15^ The Moss *et al.*, 2024 samples had Fe concentrations identical to the NCS sample Fe concentration used in this study.

We also conducted zeta potential measurements of biotin-PEG_4_-MNPs suspended in 1× PBS and 50% FBS (Table 1). The biotin-PEG_4_-MNPs had zeta potential of −1.24 ± 13.6 mV and −8.34 ± 5.3 mV, while suspended in 1× PBS and 50% FBS, respectively. Biotin-PEG_4_-MNP zeta potential while suspended in 1× PBS did not statistically significantly differ from biotin-PEG_4_-MNP zeta potential while suspended in 50% FBS (95% threshold). Our zeta potential measurement for biotin-PEG_4_-MNPs suspended in 1× PBS had a relatively large standard deviation of 13.61 mV. Wherein the zeta potential data oscillated about 0 mV. Similarly, Pamies *et al.*, 2014 observed zeta potential oscillations about 0 mV for NPs suspended in diluent with electrolyte concentrations ≥0.07 M.^36^

We measured the *D*_Hyd_ of biotin-PEG_4_-MNPs suspended in 0% (1× PBS), 10%, 20%, 30%, 40%, and 50% FBS solutions. We did not observe a linear relationship between biotin-PEG_4_-MNP *D*_Hyd_ and diluent off-target serum protein concentrations (Fig. 6 and Table 1). The *D*_Hyd_ for biotin-PEG_4_-MNPs suspended in 0% (1× PBS), 10%, 20%, 30%, 40%, and 50% FBS solutions were 62 ± 1 nm, 59 ± 3 nm, 66 ± 4 nm, 65 ± 5 nm, 66 ± 3 nm, and 63 ± 5 nm, respectively. Biotin-PEG_4_-MNP *D*_Hyd_ while suspended in a 10% FBS solution statistically significantly differed (99.67% threshold) from biotin-PEG_4_-MNP *D*_Hyd_ while suspended in a 40% FBS solution. All other variations in biotin-PEG_4_-MNP *D*_Hyd_ with respect to diluent serum protein concentration were statistically insignificant (99.67% threshold). We do not think the statistically significant variation in biotin-PEG_4_-MNP *D*_Hyd_ while suspended in 10% *versus* 40% FBS solutions was physically meaningful. The biotin-PEG_4_-MNPs had polydispersity indices (PDIs) of 0.2 ± 0.0, 0.2 ± 0.0, 0.2 ± 0.0, 0.2 ± 0.0, 0.1 ± 0.0, and 0.2 ± 0.0 while suspended in 0% (1× PBS), 10%, 20%, 30%, 40%, and 50% FBS solutions, respectively. There were no statistically significant variations amongst biotin-PEG_4_-MNP PDI with respect to diluent off-target serum protein concentrations (99.67% threshold).

## 4 Discussion

In this work, we leveraged off-target serum proteins to increase the resolution of an fMNP-based aggregation assay. The resolution of our biotin-PEG_4_-MNP-based aggregation assay changed from 64.43 nM streptavidin in 1× PBS (which was void of off-target serum proteins) to 3.22 nM streptavidin in 50% FBS (which had off-target serum protein concentrations similar to those found *in vivo*). Our biotin-PEG_4_-MNPs were biotinylated SHA 25 MNPs. The physical and magnetic properties of SHA 25 MNPs have been thoroughly characterized in the literature.^37^ We were capable of measuring streptavidin concentrations ranging from 4.83 nM to 14.50 nM with a 3.22 nM resolution in 50% FBS. Our sensing capabilities were limited in 1× PBS. Wherein, we were only capable of measuring streptavidin concentrations ranging from 0 nM to 96.65 nM with a 64.43 nM resolution. The Caruso research group at The University of Melbourne similarly demonstrated increases in fNP targeting efficiency after protein corona formation.^38,39^ Dai *et al.*, 2015 functionalized polymer coated silica NPs with anti HER2 affibodies to lend them an affinity for human ovarian cancer cells. Next, the polymer coated silica-anti-HER2-affibody (anti-HER2-SiO_2_) NPs were exposed to either a human serum (HS) solution or a human serum albumin (HSA) solution, wherein the NPs developed an HS derived corona or an HSA derived corona, respectively. Human ovarian cancer cell targeting increased from ∼70% for pristine anti-HER2-SiO_2_ NPs, to ∼90% for HSA corona anti-HER2-SiO_2_ NPs.^38^ The HSA corona anti-HER2-SiO_2_ NPs had a higher affinity for human ovarian cancer cells than pristine anti-HER2-SiO_2_ NPs. Moreover, human ovarian cancer cell targeting decreased from ∼70% for pristine anti-HER2-SiO_2_ NPs to ∼7% for HS corona anti-HER2-SiO_2_ NPs.^38^ Therefore, the HSA corona increased target affinity, while the HS corona decreased target affinity. The increase in human ovarian cancer cell targeting for the HSA corona anti-HER2-SiO_2_ NPs was not a result of HSA-cell interactions. Rather, HSA corona anti-HER2-SiO_2_ NPs exhibited higher accessibility for affibody-cell receptor binding compared to the pristine and HS corona anti-HER2-SiO_2_ NPs.^38^

In this work, since the low affinity off-target protein diluent (1 µM BSA solution) impacted biotin-PEG_4_-MNP streptavidin sensitivity in a similar manner as the off-target proteins present in 50% FBS, we can conclude that competition with low affinity off-target protein populations strongly contributed to the change in assay resolution from 64.43 nM streptavidin in 1× PBS (in the absence of off-target proteins) to 3.22 nM streptavidin in 50% FBS (in the presence of off-target proteins). Conversely, biotin-PEG_4_-MNP streptavidin sensitivity substantially decreased while suspended in the high affinity off-target protein diluent (1 µM mono streptavidin solution). We attribute increases in assay sensitivity while in the presence of a low affinity off-target protein population *versus* decreases in assay sensitivity while in the presence of a high affinity off-target protein population to the rationale underpinning competitive enzyme-linked immunosorbent assays (cELISA). Hua *et al.*, 2015 reported a higher sensitivity and wider dynamic range for a cELISA compared to a non-competitive ELISA.^40^ The competitive reaction efficiency (CE) for cELISA can be modeled with^41^
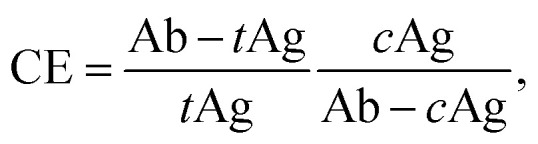
where *t*Ag, *c*Ag, Ab–*t*Ag, and Ab–*c*Ag are the concentrations of the target antigen, competing antigen, antibody-target antigen complex, and antibody-competing antigen complex at equilibrium, respectively. This expression suggests that competitive assays are most efficient when there is a high concentration of a low affinity competitor in the sensing environment.^41^ Several researchers have demonstrated an increase in competitive assay sensitivity *via* a decrease in competitor binding affinity.^42–44^ Xiong *et al.*, 2017 increased the sensitivity of a direct competitive fluorescence-linked immunosorbent assay (dcFLISA) by decreasing the competitor-antibody binding affinity.^42^

Additionally, we demonstrated the time evolution of biotin-PEG_4_-MNP *D*_Hyd_ in 1× PBS and 50% FBS at room temperature. The biotin-PEG_4_-MNPs suspended in 1× PBS underwent substantial *D*_Hyd_ increases at hour 2 of the 8-hour incubation. Conversely, the biotin-PEG_4_-MNPs suspended in 50% FBS remained stable throughout the duration of the 8-hour room temperature incubation. Our data suggests that the presence of off-target serum proteins belonging to the 50% FBS solution stabilized the biotin-PEG_4_-MNPs against aggregation for 8 hours at room temperature. These data are in alignment with MNP-protein corona complex literature, which suggests that serum proteins are capable of stabilizing MNP suspensions against aggregation.^14,28–30^ We used 70 µL 80 mg Fe per L DLS samples to collect these data. In a previous work, we used 1 mL 10 mg Fe per L DLS samples to monitor the time evolution of biotin-PEG_4_-MNP *D*_Hyd_ at room temperature, and found that the biotin-PEG_4_-MNPs were stable for the duration of the 8-hour incubation.^15^ Our previous result seemingly contradicts our observation of substantial biotin-PEG_4_-MNP *D*_Hyd_ increases at hour 2 of their 8-hour incubation in 1× PBS. We attribute the difference in biotin-PEG_4_-MNP stability at room temperature in 1× PBS, to differences in biotin-PEG_4_-MNP concentration. MNP *D*_Hyd_ measurements can be quite sensitive to sample Fe concentration.^45,46^ Lim *et al.*, 2013 demonstrated a ∼148 nm increase in MNP *D*_Hyd_ due to an increase in the sample solution's Fe concentration from 100 to 250 mg Fe per L for 18 nm superparamagnetic MNPs.^45^ Our more highly concentrated time evolution DLS sample (80 mg Fe per L) became unstable at hour 2, while the lesser concentrated sample from Moss *et al.*, 2024 (10 mg Fe per L) remained stable for the entirety of the 8-hour room temperature incubation. Non-target induced fMNP aggregation can be modeled with the extended Derjaguin–Landau–Verwey–Overbeek (xDLVO) theory. Wherein, fMNP aggregation is dependent on the sum of the repulsive electrostatic double layer (EDL) force and the attractive van der Waals and dipolar forces.^27,47–49^ According to xDLVO theory models, attractive van der Waals force dominated interactions amongst fMNPs are more likely at shorter interparticle distances. fMNPs suspended in more highly concentrated samples are in closer proximity, compared to fMNPs suspended in lower concentrated samples. Therefore, our observation of increased non-target induced aggregation in the 80 mg Fe per L 1× PBS biotin-PEG_4_-MNP sample, compared to the 10 mg Fe per L 1× PBS biotin-PEG_4_-MNP sample, can be explained with the xDLVO theory.

We also monitored biotin-PEG_4_-MNP *D*_Hyd_ with respect to diluent FBS contents to gauge the extent of protein corona formation with increasing diluent off-target serum protein concentrations. Our measurements were acquired shortly after the biotin-PEG_4_-MNPs were exposed to each solution. NPs can undergo substantial *D*_Hyd_ increases directly after exposure to protein rich media.^12,13^ For example, Barran *et al.*, 2013 reported a 120 nm increase in lipid NP *D*_Hyd_ after a 1-minute 37 °C incubation in a human plasma solution.^13^ Moreover, Casals *et al.*, 2010 observed an 18 nm increase in gold NP (AuNP) *D*_Hyd_ after a minutes long 37 °C incubation in cell culture medium supplemented with serum.^12^ The majority of NP-protein corona complex studies are geared toward *in vivo* applications and are conducted at physiological temperature (∼37 °C).^8,9,12,13,18,19,24^ Our 1× PBS and 50% FBS streptavidin-response curves were prepared and maintained at room temperature (∼25 °C). Therefore, we are interested in gauging the extent of protein corona formation with increasing diluent serum protein concentrations at room temperature (∼25 °C). Mahmoudi *et al.*, 2013 demonstrated the effects of incubation temperature on iron oxide NP protein corona thickness and composition.^7^ Their results suggest that serum protein adsorption was maximal at 40 °C, 43 °C, and 37 °C for iron oxide NPs with positive, neutral, and negative surface charges, respectively.^7^ The identity and prevalence of adsorbed proteins were monitored at temperatures spanning from 5 °C to 45 °C. Therefore, the levels of protein adsorption reported here may be less pronounced compared to studies outlined in the literature, given that serum protein adsorption may be less prominent at room temperature. It is also important to note that the extent of non-specific serum protein adsorption is not solely reliant on diluent serum protein concentrations,^14^ it is also reliant on NP size, shape, and surface charge as well as environmental conditions. Hence, it is difficult to predict the relationship between biotin-PEG_4_-MNP *D*_Hyd_ and serum protein concentrations, given the many variables that contribute to non-specific NP–protein interactions.

The *D*_Hyd_ data for biotin-PEG_4_-MNPs suspended in 0% (1× PBS), 10%, 20%, 30%, 40%, and 50% FBS solutions suggest there was no correlation between biotin-PEG_4_-MNP *D*_Hyd_ and diluent off-target serum protein concentrations. Wherein there were minimal variations in biotin-PEG_4_-MNP *D*_Hyd_ with respect to diluent off-target serum protein concentrations. However, we suspect the biotin-PEG_4_-MNPs and serum proteins were interacting given substantial increases in biotin-PEG_4_-MNP sensitivity to streptavidin and decreases in biotin-PEG_4_-MNP zeta potential while suspended in 50% FBS, compared to 1× PBS. The off-target serum proteins present in 50% FBS effected our biotin-PEG_4_-MNPs such that their ϕ_3_ and *D*_Hyd_ in a 97 nM streptavidin 1× PBS solution were statistically indistinguishable from their *ϕ*_3_ and *D*_Hyd_ in an 18 nM streptavidin 50% FBS solution. These results suggest a substantial increase in biotin-PEG_4_-MNP streptavidin sensitivity in 50% FBS, compared to 1× PBS. Moreover, biotin-PEG_4_-MNPs underwent a statistically insignificant decrease in zeta poteintial from −1.2 ± 13.6 mV in 1× PBS to −8.3 ± 5.3 mV in 50% FBS. We think such increases in biotin-PEG_4_-MNP streptravidin sensitivity and decreases in biotin-PEG_4_-MNP zeta potential were due to the adsorption of off-target serum proteins to the biotin-PEG_4_-MNPs. Other factors can cause an increase in biotin-PEG_4_-MNP streptavidin sensitivity, including decreases in biotin-PEG_4_-MNP zeta potential and increases in diluent salt content.^15^ It is unlikely that changes in biotin-PEG_4_-MNP zeta potential caused the increase sensitivity to streptavidin, given that the absolute value of biotin-PEG_4_-MNP zeta potential increased in 50% FBS (although such increases in zeta potential absolute value were statistically insignificant). According to the xDLVO theory, an increase in fMNP zeta potential absolute value would limit fMNP aggregation and lead to a decrease in target sensitivity.

It is also possible that the protein corona surrounding the biotin-PEG_4_-MNPs was too thin for detection *via* DLS measurement. Jans *et al.*, 2009 suggests that a complete layer of adsorbed protein can increase NP *D*_Hyd_ by 2 times the protein diameter.^50^ Bovine serum albumin (BSA) is the most prevalent protein in FBS^14,31^ and its hydrodynamic radius has been reported as 3.8 nm^28^ and 4.6 nm.^51^ Moreover, Casals *et al.*, 2010 observed a 3 nm thick BSA monolayer on AuNPs.^12^ Therefore, we estimate that serum protein adsorption could have increased biotin-PEG_4_-MNP *D*_Hyd_ by ∼6 nm to ∼20 nm. However, to distinguish between particle size populations with DLS, the particles must differ in size by a factor of 3 (for example, 10 nm *versus* 30 nm).^46^ Otherwise, one will obtain a size intensity distribution with a relatively broad peak with a larger PDI.^46^ Therefore, a ∼6 nm to ∼20 nm increase in biotin-PEG_4_-MNP *D*_Hyd_ would be unobservable *via* DLS measurement.

## 5 Conclusions

Our work shows that (1) the resolution of an fMNP-based aggregation assay changed from 64.43 nM streptavidin in 1× PBS to 3.22 nM streptavidin in 50% FBS. In 1× PBS our aggregation assay had a dynamic range spanning from 0 to 96.65 nM streptavidin with a resolution of 64.43 nM streptavidin. In 50% FBS, the dynamic range of our aggregation assay shifted to lower streptavidin concentrations. Wherein, our aggregation assay had a dynamic range spanning from 4.83 nM streptavidin to 14.50 nM streptavidin with a resolution of 3.22 nM streptavidin. We attribute the increase in biotin-PEG_4_-MNP streptavidin sensitivity to competition between streptavidin and off-target serum proteins with a low biotin binding affinity. However, competition with a high affinity off-target protein population decreased streptavidin sensitivity. We attribute increases in assay sensitivity while in the presence of a low affinity off-target protein population *versus* decreases in assay sensitivity while in the presence of a high affinity off-target protein population to the rationale underpinning cELISA. (2) Biotin-PEG_4_-MNPs suspended in 1× PBS underwent significant *D*_Hyd_ increases at hour 2 of an 8-hour room temperature incubation, while biotin-PEG_4_-MNPs suspended in 50% FBS did not undergo substantial *D*_Hyd_ fluctuations for the entirety of an 8-hour room temperature incubation. These data suggest that the off-target serum proteins present in 50% FBS solutions stabilized biotin-PEG_4_-MNPs against aggregation for at least 8 hours at room temperature. Further research is required to study changes in fMNP-based assay Δ*ϕ*_3_ for a multivalent target *versus* a monovalent target. Our data suggests that the mono-streptavidin corona triggered a larger Δ*ϕ*_3_ than streptavidin induced aggregation. Which suggests that fMNP-based assays targeting monovalent biomolecules can possess comparable or greater sensitivity than fMNP-based assays targeting multivalent biomolecules.

## Author contributions

Gabrielle Moss: conceptualization; methodology; investigation; validation; visualization; writing – original draft preparation. Christian Knopke: conceptualization; methodology; writing – review and editing. Solomon G. Diamond: conceptualization; supervision; writing – review and editing.

## Conflicts of interest

Authors Christian Knopke and Solomon G. Diamond were employed by and hold ownership interests in the company Lodestone Biomedical Inc. The remaining authors declare that the research was conducted in the absence of any commercial or financial relationships that could be construed as a potential conflict of interest.

## Data Availability

Data for this article, including raw magnetic particle spectroscopy data and dynamic light scattering data are available on Figshare at https://figshare.com/authors/Gabrielle_Moss/19742920.
